# Supplementary material for: A new surgical tape with a mesh designed to prevent skin tears and reduce pain during tape removal
Source: PLoS One. 2023 Jul 10;18(7):e0288304. doi: 10.1371/journal.pone.0288304 (PMC10332619; doi:10.1371/journal.pone.0288304)
Supplement: Attachment — Submitted filename: PONE-D-22-33348R1_comments.pdf [file pone.0288304.s004.pdf]

The article “A new surgical tape with a mesh designed to prevent skin tears and reduce pain during tape removal” has provided emphasized on considerable topic. The surgical tapes are very painful when taken off after the surgery. This seems to be very innovative idea to put mesh in between the skin and the tape.

Here are some questions for the authors;

1. Did the authors find any kind of side effect of mesh on the skin?
2. Does this tape removal need any expertise or any other person can do it?
3. Does this tape is cost effective?
4. Did authors compared this method with the other different approaches (under clinical trial) for preventing medical adhesive-related skin injury?
