# Supplementary material for: A new surgical tape with a mesh designed to prevent skin tears and reduce pain during tape removal
Source: PLoS One. 2023 Jul 10;18(7):e0288304. doi: 10.1371/journal.pone.0288304 (PMC10332619; doi:10.1371/journal.pone.0288304)
Supplement: Attachment — Submitted filename: Comment_PONE-D-22-33348R2.pdf [file pone.0288304.s009.pdf]

The authors of the article “A new surgical tape with a mesh designed to prevent skin tears and reduce pain during tape removal” has included all the comments raised in the manuscript and explained them properly.

The manuscript can be considered for the publication in “PLOS One”.
