# Supplementary material for: A new surgical tape with a mesh designed to prevent skin tears and reduce pain during tape removal
Source: PLoS One. 2023 Jul 10;18(7):e0288304. doi: 10.1371/journal.pone.0288304 (PMC10332619; doi:10.1371/journal.pone.0288304)
Supplement: Attachment — Submitted filename: Responses to Reviewers.docx [file pone.0288304.s007.docx]

POINT-BY-POINT RESPONSES TO REVIEWERS

**This does not alter our adherence to PLOS ONE policies on sharing data and materials.**

**REVIEWER #1:**

**(Comment 1)**

*The article “A new surgical tape with a mesh designed to prevent skin tears and reduce pain during tape removal” has provided emphasized on considerable topic. The surgical tapes are very painful when taken off after the surgery. This seems to be very innovative idea to put mesh in between the skin and the tape. Here are some questions for the authors.*

**(Response)** Thank you for pointing out the problem of pain during tape removal. And thank you very much for scrutinizing our manuscript. According to your kind suggestions, we revised the manuscript. They were very helpful to improve its quality. We would appreciate your re-consideration of our revised paper.

**(Comment 2)**

*Did the authors find any kind of side effect of mesh on the skin?*

**(Response)**

Thank you very much for important question about side effect of mesh on the skin. Our mesh is made from olefin and was made to be as thin as possible while still maintaining its stiffness. But the mesh caused prickly skin irritation and reticulated skin deformation after application (although they recovered without problems after some minutes). (in the Discussion section on page 15, lines 333 to 334)

We are still working on improving the tape. Changing the base material from non-woven fabric to film improved the tape's ability to conform to the skin. As a result, there is less fine movement of the mesh between the skin and the base material, reducing skin irritation.

Therefore, we added the following sentences in the Discussion section on page 16, lines 337 to 340: “By using a thin and flexible film as the tape substrate and improving the tape's ability to conform to the skin, it may be possible to obtain strong adhesion even with mesh and reduce itchy skin irritation caused by the mesh.”

**(Comment 3)**

*Does this tape removal need any expertise or any other person can do it?*

**(Response)**

Thank you for pointing out the tape usability that is important task in the developing new tape. The product should be designed so that even nurses with presbyopia can easily remove the tape. We have added the following sentences in the Discussion section on page 16 lines 340 to 343: “Improvements in such as biocompatibility, manufacturing cost, tape design that anyone can use are expected to lead to products that surpasses silicon taping and the work of peeling off with a remover in clinical site.”

**(Comment 4)**

*Does this tape is cost effective?*

**(Response)**

Thank you for the question about the production cost. We are developing the new tape that is cheaper than or equal to silicon tape. We have added the following sentences in the Discussion section on page 16, lines 340 to 343: “Improvements in such as biocompatibility, manufacturing cost, tape design that anyone can use are expected to lead to products that surpasses silicon taping and the work of peeling off with a remover in clinical site.”

**(Comment 5)**

*Did authors compared this method with the other different approaches (under clinical trial) for preventing medical adhesive-related skin injury?*

**(Response)**

We are sorry that we have not yet performed a comparative analysis of other approaches and our mesh method for preventing medical adhesive-related skin injury. Each method probably has advantages and disadvantages, and it may be difficult to scientifically judge their superiority.

**REVIEWER #2:**

**(Comment 1)**

*The manuscript is technically sound and all the data mentioned in the manuscript are supporting the conclusions. Statistical data are analyzed appropriately and clearly. All the data mentioned in the manuscript are available in the literature. The manuscript is presented in an intelligent fashion and written in standards English. The correction may kindly be made as per the reviewed manuscript.*

**(Response)**

Thank you for your positive comments on our manuscript. We feel honored.

**REVIEWER #3:**

**(Comment 1)**

*The description of new tape in terms of the materials and what properties been considered for its selection for study need to be thoroughly introduced in the Introduction.*

**(Response)**

Thank you very much for scrutinizing our manuscript. According to your important questions and suggestions, we revised the manuscript.

We have added the following sentences to introduce the experiment tape in the Introduction section on page 3, lines 58 to 62: “A tape manufacturer combined mesh with the medical tape used to secure dialysis routes in hospitals to create the experimental tape. We attempted to clarify the advantages and disadvantages of mesh by adding no new components of medical tape other than mesh in this experiment.”

**(Comment 2)**

*Details of the substrate and adhesive used in the study need to be more elaborated.*

**(Response)**

Thank you for pointing out the lack of information about the experimental tapes. Since the properties of mesh has already been described in the manuscript, the description of the tape substrate, the adhesive, and their processing method has been added in the Material and Methods section on page 4, line 75 to 79: “Kino white (Nitoms, Inc., Tokyo, Japan) was used as the tape substrate and the adhesive of the experimental tape. Kino white is a highly adhesive medical tape composed of non-woven fabric and acrylic adhesive, and is used in dialysis hospitals to fix tubes to the patient's skin. The experimental tape was made by a tape manufacturer (Cosmotec Co., Ltd.) by combining Kino white and olefin mesh.”

**(Comment 3)**

*The manuscript is lacking tables and figures.*

**(Response)**

Thank you for reviewing our manuscript despite the lack of figures. The figures were submitted to the editors on the web. Sorry for the inconvenience, we kindly ask to request figures from the editor.

**(Comment 4)**

*Statistical data and the results are not provided.*

**(Response)**

Thank you for your comment. The results of the statistical analysis as figures 6, 7, and 8 have been submitted to the editor on the web. We kindly ask to request the figures from the editor. And we will additionally submit measurement results of Degree of pain as a supplemental table, so please check and evaluate the table.

**(Comment 5)**

*Language is scientific with clarity of expression.*

**(Response)**

Thank you for your comment.

**(Comment 6)**

*Please make sure that words are separated by a space.*

**(Response)**

Thank you for pointing out our mistakes. We made several corrections as below points: Page 2, line 33, 35, 39., Page 6, line 115, 127., Page 7, line 132., Page 8, line 169., Page 10, line 214., Page 11, line 223., Page14, line 308., Page 19, line 401.

**(Comment 7)**

*Reference line 363 author name has been corrected 'Lamers E, van Kempen THS, Baaijens, FPT, Peters GWM, Oomens CWJ'.*

**(Response)**

Thank you for pointing out the mistake. We made corrections as below points in the References section on page 17, line 371 to 373: “1. Lamers E, van Kempen THS, Baaijens FPT, Peters GWM, Oomens CWJ. Large amplitude oscillatory shear properties of human skin. J Mech Behav Biomed Mater. 2013;28: 462-470. doi:10.1016/j.jmbbm.2013.01.024.”
